# Impact of COVID-19 pandemic on surgical activity in the Brazilian private healthcare system

**DOI:** 10.1371/journal.pone.0289032

**Published:** 2023-12-14

**Authors:** Luiza Helena Degani Costa, Barbara Yepes Pereira, Isabela Queiros Castro, Heitor Werneck, Glenio B. Mizubuti, Luiz Fernando dos Reis Falcão

**Affiliations:** 1 Faculdade Israelita de Ciências da Saúde Albert Einstein, Hospital Israelita Albert Einstein, São Paulo, São Paulo, Brazil; 2 Centro Universitário São Camilo, São Paulo, São Paulo, Brazil; 3 Department of Biostatistics, Hospital Israelita Albert Einstein, São Paulo, Brazil; 4 Agência Nacional de Saúde Suplementar, Rio de Janeiro, Rio de Janeiro, Brazil; 5 Kingston Health Sciences Center, Queen’s University, Kingston, Ontario, Canada; 6 Universidade Federal de São Paulo – Escola Paulista de Medicina (UNIFESP-EPM), São Paulo, São Paulo, Brazil; IRCCS Burlo Garofolo Trieste, ITALY

## Abstract

**Introduction:**

Surgical volume was drastically reduced in many countries due to challenges imposed by the COVID-19 pandemic.

**Objectives:**

We sought to estimate the number of cancelled surgical and diagnostic procedures within the Brazilian private healthcare system between 2020 and 2021 over the course of the COVID-19 pandemic, and to project the procedural backlog generated for specific elective and time-sensitive surgeries, and diagnostic procedures.

**Methods:**

Data were systematically extracted from the Brazilian national regulatory agency for the private healthcare system and included (i) quarterly and annual surgical and diagnostic volume, and (ii) the number of private health insurance beneficiaries between January 2016 and June 2021. Based on pre-pandemic data we estimated the expected number of surgical and diagnostic procedures that failed to be performed between 2020 and 2021.

**Results:**

The average quarterly surgical and diagnostic procedures declined by 29.5% in 2020 and by 21.5% in 2021 compared to 2019. In 2020, such reduction reflected a lower number of diagnostic procedures under anesthesia (-35.1%), as well as elective (-14.7%), time-sensitive (-18.8%), and urgent (-4.6%) surgeries. In the first half of 2021, though the surgical and diagnostic procedures increased compared to 2020, they remained significantly below their historical average. The estimated backlogs were 134.385,64 for total surgical procedures, 2.634,64 for bariatric surgery and arthroplasty revision (elective surgeries), 2.845,61 for oncologic (time-sensitive) surgeries, and 304.193,99 for diagnostic procedures, requiring 1.7, 15.9, and 6.8 years, respectively, to make up for such backlogs.

**Conclusion:**

There was a major decline on the number of surgical and diagnostic procedures due to the COVID-19 pandemic. Despite a slight recovery of elective surgeries throughout the pandemic, many time-sensitive surgeries and diagnostic procedures were cancelled, with potential medium- to long-term consequences to patients and the system as a whole.

## 1. Introduction

The COVID-19 pandemic has directly affected the organization of healthcare systems and their capacity to respond to existing (and rapidly increasing) demands. In this context, even healthcare systems considered to be resilient and efficient struggled to deal with the magnitude of the shock caused by the pandemic [1].

In Brazil, the first case of SARS-CoV-2 was confirmed in February 2020 [2]. At the time, the lack of knowledge about the disease, combined with limited resources (e.g., personal protection equipment (PPE), supplementary oxygen, and mechanical ventilators), and the lack of coordinated public health policies generated significant barriers to the maintenance/functionality of the healthcare system [3–5]. Accordingly, the impact on the overall surgical volume was substantial. The COVIDSurg study estimated that nearly 3 million surgeries were cancelled in Brazil between January and April of 2020 [6]. As the pandemic persisted, population-based studies confirmed its major impact on the Brazilian public healthcare system (SUS). Bigoni et al. [3] evaluated the volume of ambulatory, diagnostic and screening procedures, oncologic treatment, dialysis, transplant, surgical and obstetric procedures in 2020, and demonstrated an overall 25% reduction compared to 2019. In that sense, Truche et al. [4] estimated that between 675,202 and 1,208,769 elective surgeries were cancelled in Brazil in 2020 in the *public* healthcare system alone.

It is estimated that ~75% of the Brazilian population relies solely on the public healthcare system, whereas the remaining 25% have access to private health insurance [7]. Notably, important issues commonly observed in Brazilian public hospitals (e.g., lack of hospital beds and/or minimal resources to ensure adequate patient management) are generally not observed in the private sector, although financial sustainability of the private system has been a subject of debate due to the ever-growing healthcare costs [8]. Nevertheless, given the significant differences between the Brazilian public and private health systems, any attempt at analysing the impact of the pandemic on the national surgical volume will remain incomplete without a thorough assessment of the private healthcare system.

In March 2020, the Brazilian National Regulatory Agency for the private healthcare system (Agência Nacional de Saúde Suplementar–ANS) enforced a policy that postponed/cancelled all elective surgical procedures to ensure the safety of patients and healthcare professionals, as well as to preserve the already scarce resources needed for COVID-19 patients [9]. While necessary from an epidemiologic standpoint, this decision not only directly impacted the health and quality of life of patients suffering from chronic illnesses [10, 11], but it also severely affected the financial balance of several stakeholders in the private healthcare sector [12]. The resulting surgical backlog combined with the sector’s operational efficiency (or lack thereof) will, therefore, determine the short-, mid-, and long-term consequences for the sector and its pool of beneficiaries.

The primary objective of the present investigation was to estimate the total number of surgeries and diagnostic procedures under anesthesia (bronchoscopies, upper endoscopies, and colonoscopies) that were cancelled due to the pandemic in the Brazilian supplementary (private) healthcare sector between 2020 and 2021. Secondary objectives included (i) to determine the impact of the pandemic on each surgical category (i.e., elective, time-sensitive, and urgent); and (ii) to estimate the time required to compensate for the surgical backlog generated by the pandemic.

## 2. Methods

### 2.1 Study design and datasets

This is a retrospective, population-based study. Data were systematically extracted from ANS, as well as an independent organization (Global Change Data Lab), and were freely accessible to the public. Hence, ethics approval and patients’ consent were waived by the Institutional Research Ethics Board.

Data from the supplementary healthcare system originated from different datasets that fed into the Power BI (Microsoft Power Platform, Microsoft^®^, USA) of the ANS’s official website(https://app.powerbi.com/view?r=eyJrIjoiZDFkODkxNzMtODgwNC00ZTFiLTg2MzUtZmEwNDViNmU1ZWI4IiwidCI6IjlkYmE0ODBjLTRmYTctNDJmNC1iYmEzLTBmYjEzNzVmYmU1ZiJ9), which aggregates quarterly and annual volumes of selected clinical, surgical, dental health and diagnostic appointments and admissions performed in the Brazilian supplementary healthcare system, as well as its quarterly number of beneficiaries. From the dataset of healthcare system productivity, all data collected between 2016 and 2021 on (i) total surgical admission; (ii) obstetric procedures; (iii) femur fracture repair; (iv) bariatric surgery; (v) arthroplasty revision; (vi) breast cancer surgery; (vii) uterine cervical cancer surgery; (viii) colon and rectal cancer surgery; (ix) prostate cancer surgery; (x) bronchoscopy; (xi) upper endoscopy/esophagogastroduodenoscopy, and (xii) colonoscopy were included. From the dataset of insurance beneficiaries, we extracted quarterly number of beneficiaries recorded between 2016 and 2021. Individuals younger than 15 years old were then excluded from the analysis, as this is a population who is very unlikely to need any of the specific procedures included in our analysis.

The incidence of COVID-19 in Brazil was calculated based on the official data available on the online platform *Our World in Data* (https://ourworldindata.org/covid-cases), with the absolute number of new COVID-19 cases presented in quarterly aggregates. This allowed us to characterize the epidemiologic status of the pandemic in each quarter to put into perspective the productivity data. Data presented by *Our World in Data* are open access under the *Creative Commons BY* license. The codes and software are open coded and available via GitHub under the MIT permissive license. The specific COVID-19 data source is CSSE COVID-19 from the Johns Hopkins University.

The time series was divided into 3 intervals: (1) baseline or pre-pandemic, from January 2016 to December 2019; (2) pandemic 1, from January to December 2020; and (3) pandemic 2, from January to June 2021. While the COVID-19 pandemic did not end in 2020, the public health policies pertaining to elective surgical and diagnostic procedures in 2021 differed from the previous year–partly due to the ample availability of diagnostic tests for SARS-CoV-2, as well as some flexibility on the imposed measures of social isolation–thereby allowing the resumption of elective diagnostic and surgical procedures in 2021.

To better understand the impact of the pandemic in different surgical contexts, the analysed procedures were further grouped as “urgency and emergency”, including the obstetric procedures and femur fracture repairs; “elective”, including bariatric and arthroplasty revision procedures; “time-sensitive”, including cancer (breast, uterine cervix, colon, rectum, and prostate) procedures; and “ambulatory diagnostic procedures”, including bronchoscopies, upper endoscopies, and colonoscopies.

### 2.2 Statistical analysis

The number of surgical/diagnostic procedures were extracted and analyzed in a quarterly fashion, from January 2016 to June 2021 (total of 22 quarters). For each studied category, the ratio $\frac{number\;of\;procedures}{number\;of\;beneficiaries}\;x\;1000$ was calculated for each quarter. The pre-pandemic data (January 2016- December 2019) were used to determine the estimated number of surgical/diagnostic procedures that were cancelled in the Brazilian supplementary healthcare system in 2020 and 2021 (total backlog), as well as procedural backlogs specific to each category (elective, urgent, and time-sensitive surgeries) according to the annual growth rate of the sector.

Initial analyses included graphic and temporal decomposition to identify any potential seasonal tendencies. Inferential tests (i.e., ADF, KPSS e DF-GLS) were performed to verify stationarity and graphical autocorrelation. Either Holt-Winters exponential smoothing or Seasonal Autoregressive Integrated Moving Average (SARIMA) models applied to the pre-pandemic temporal series were used to estimate the procedural volumes for 2020 and 2021 had the pandemic not occurred. Both Holt-Winters and SARIMA allow to model series with seasonal tendency [13, 14], so both were initially applied to each of the temporal series and the best model fit was ultimately chosen based on graphical analysis (Ljung-Box test, autocorrelation function plot of model residuals, histogram of residuals and standardized residuals) and error measures. Additionally, we performed a stratified analysis according to the type of urgent surgical procedure following the same (above-mentioned) analytical strategy in order to better understand the drivers of their tendencies and trajectories throughout the studied period.

Upon estimating the expected quarterly numbers of total surgical and diagnostic procedures for 2020 and 2021, we then estimated the total procedural backlog for each analyzed quarter of 2020 and 2021 based on the difference between the values derived from the temporal models and those obtained from the ANS’s database. Following the same strategy, backlogs for elective and time-sensitive surgeries were also estimated. Backlogs were analysed using generalized linear models with gamma distribution, considering the interval and procedural type as covariates, and the results were presented using coefficients, CIs, and p-values. Finally, the following equation was used to estimate the time required to make up for such backlogs, with results presented in quarters and years: *Backlog = 2*,576 + Procedural type coefficient + procedural type coefficient * (period/interval).

Statistical analyses were performed using R Stats Package version 3.4 (Copyright Mayo Foundation for Medical Education and Research, 2022) and IBM Statistical Package for the Social Sciences (SPSS, version 26.0 for Windows, Armonk, New York 2019). A p-value of <0.05 was be used as the criteria for statistical significance.

## 3. Results

Table 1 shows the annual and quarterly number of procedures during the studied period. Notably, the total surgical activity had an increasing (~10% annually) tendency from 2016 to 2019. Subsequently, however, the overall annual surgical and ambulatory diagnostic procedures dropped by 23.6% and 35.1%, respectively, in 2020 when compared to 2019.

**Table 1:** Total surgical volume* and ambulatory diagnostic procedures quarterly** between 2016 and 2021.

		Total surgical activity	Ambulatory diagnostic procedures
2016	Q1	1,031,296	1,163,488
	Q2	1,079,268	1,190,132
	Q3	993,707	1,094,826
	Q4	940,208	1,041,262
	Annual total	4,044,479	4,489,708
2017	Q1	1,055,921	1,182,677
	Q2	1,067,888	1,207,111
	Q3	1,031,365	1,161,568
	Q4	1,010,340	1,138,117
	Annual total	4,165,514	4,689,473
2018	Q1	1,028,820	1,237,576
	Q2	1,191,541	1,261,021
	Q3	1,103,572	1,200,612
	Q4	1,056,507	1,131,075
	Annual total	4,380,440	4,830,284
2019	Q1	1,099,976	1,232,078
	Q2	1,156,008	1,245,852
	Q3	1,129,518	1,192,793
	Q4	1,063,342	1,059,055
	Annual total	4,448,844	4,729,778
2020	Q1	778,541	483,790
	Q2	826,318	754,201
	Q3	924,820	949,231
	Q4	869,170	882,255
	Anual total	3,398,849	3,069,477
2021	Q1	803,820	827,805
	Q2	978,072	992,483

*Absolute number of procedures performed in each quarter.

**Q1 = 1^st^ quarter, Q2 = 2^nd^ quarter, Q3 = 3^rd^ quarter, Q4 = 4^th^ quarter

Similarly, the average quarterly number of procedures (surgical + diagnostic) was 2,294,656 in 2019, versus 1,617,082 in 2020 and 1,801,090 in the first 2 quarters of 2021. This corresponds to a 29.5% reduction in 2020 and 21.5% in 2021 compared to 2019. While the magnitude of the impact was greater in the first half of 2020, neither ambulatory diagnostic procedures nor total surgical activity returned to their baseline levels throughout the study period (Table 1 and Appendix Table 1 in S1 Appendix).

**Fig 1:**
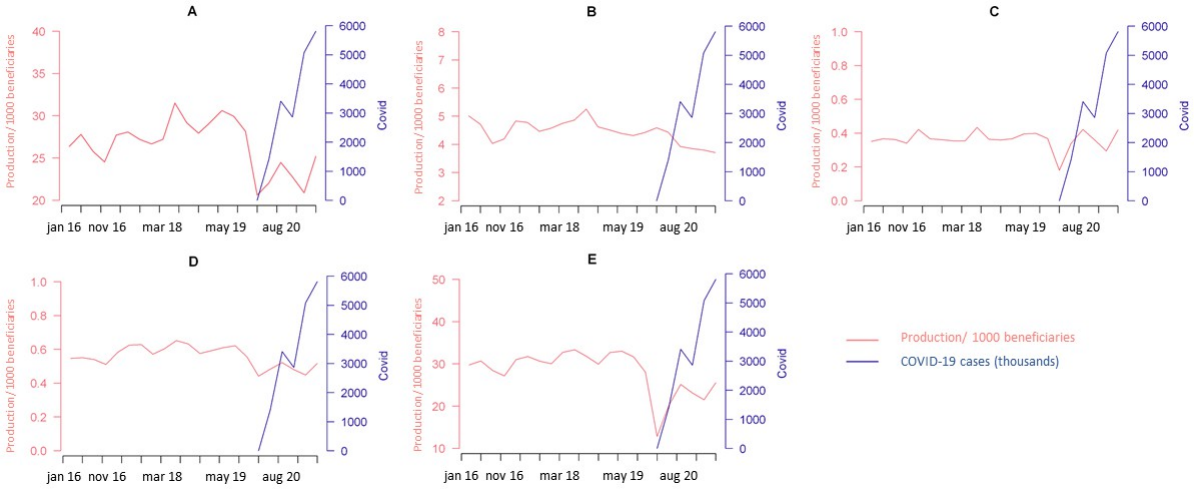
Surgical activity and ambulatory diagnostic procedures volumes (for every 1000 private insurance beneficiaries) between 2016 and 2021, and their relationship with new COVID-19 cases. A) Total surgical activity; B) Urgent surgeries; C) Elective surgeries; D) Time-sensitive surgeries; E) ADPs under anesthesia.

This is also reflected in Fig 1, which shows a graphic representation of the total surgical and diagnostic activity between 2016 and 2021, adjusted by the number of insurance beneficiaries ≥15 years-old, and their relationship with the incidence of new COVID-19 cases. Both surgical and diagnostic procedures showed a clear and marked reduction coincident with the beginning of the pandemic (Fig 1A and 1E). Additionally, while a steady increase was later observed in 2020 despite a rapid increase in COVID-19 new cases, adjusted numbers remained far below their pre-pandemic values until the end of the time series.

When focusing on ambulatory diagnostic procedures alone, the average quarterly volume declined by 35.1% in 2020 and by 23.0% in the first semester of 2021 compared to 2019. As for the surgical procedures, the average quarterly volume was 1,112,211 in 2019 and 849,712 in 2020, corresponding to a 22.6% reduction during the first pandemic period. Such reduction resulted primarily from a lower number of elective (−14.7%), time-sensitive (−18.8%), and urgent (−4.6%) surgical procedures (Fig 1 and Table 2 and Appendix Table 2 in S1 Appendix). By the end of the first semester of 2021, however, the average number of elective surgeries was back to its baseline values despite persistent increase in the incidence of COVID-19 cases, while time-sensitive surgeries remained below historical rates (Fig 1C, Table 2).

**Table 2:** Urgent, elective, and time-sensitive surgeries quarterly activity between 2016 and 2021.

		Urgent	Elective	Time-sensitive
2016	Q1	196,260	13,753	21,387
	Q2	182,968	14,197	21,348
	Q3	155,194	13,970	20,768
	Q4	160,598	13,058	19,595
2017	Q1	184,055	16,056	22,177
	Q2	181,632	13,954	23,753
	Q3	169,208	13,679	23,829
	Q4	173,184	13,399	21,621
2018	Q1	179,253	13,366	22,782
	Q2	184,038	16,416	24,651
	Q3	198,876	13,771	23,912
	Q4	174,934	13,622	21,762
2019	Q1	170,089	13,800	22,289
	Q2	165,786	14,924	23,004
	Q3	162,884	15,034	23,427
	Q4	167,001	13,873	21,012
2020	Q1	173,579	6,800	16,689
	Q2	166,131	12,720	18,119
	Q3	148,173	15,907	19,714
	Q4	147,118	13,725	18,359
2021	Q1	146,394	11,307	17,268
	Q2	144,117	16,258	20,052

*Q1 = 1^st^ quarter, Q2 = 2^nd^ quarter, Q3 = 3^rd^ quarter, Q4 = 4^th^ quarter. Note: Urgent surgeries = obstetric procedures + femur fracture; elective surgeries = bariatric surgery + arthroplasty revision; time-sensitive surgeries = breast cancer surgery + uterine cervical cancer surgery + colon and rectal cancer surgery + prostate cancer surgery.

**Fig 2:**
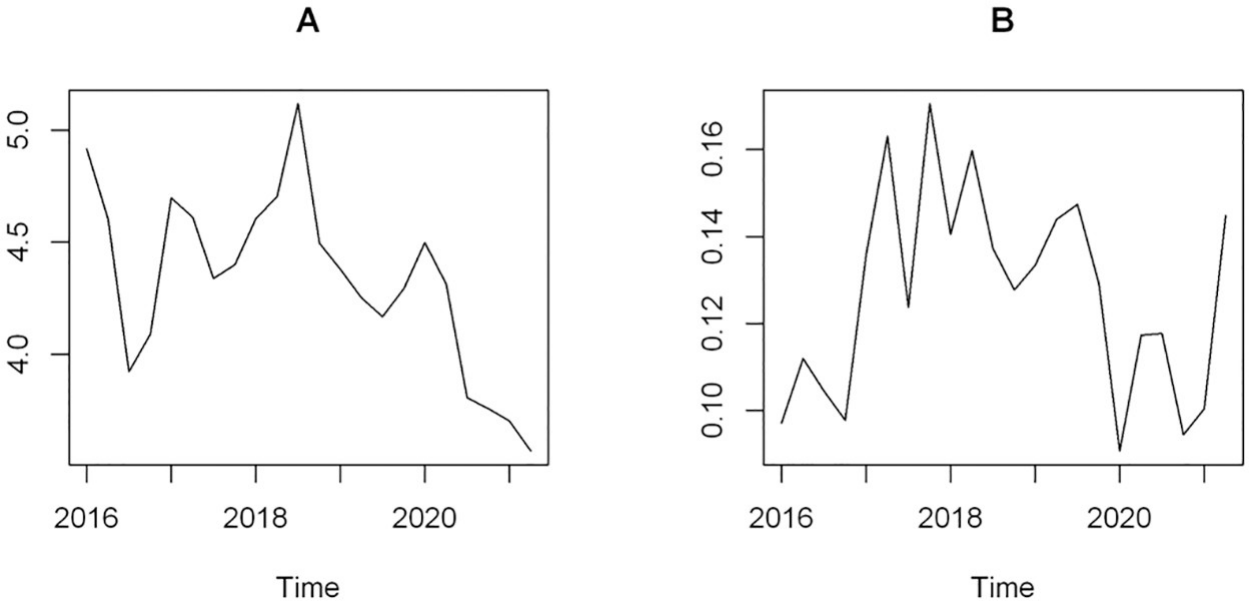
Urgent surgical procedures throughout the study period. A) Obstetric procedures; B) Femur fracture repairs.

Surprisingly, the number of urgent surgical procedures dropped significantly during the pandemic and, unlike the other surgical categories, had a progressive further reduction tendency throughout 2020 and 2021 (Fig 1B). The two components of the urgent surgery category did not show similar trends, though. While a progressive reduction was seen among obstetric procedures during the whole pandemic period (Fig 2A), femur fracture repairs reduced in the first quarter of 2020, but subsequently returned to pre-pandemic levels in the second quarter of 2021 (Fig 2B).

Table 3 shows the estimated number of procedures for each 1000 private insurance beneficiaries ≥15 years-old in 2020 and 2021 had the pandemic not occurred and based solely on the pre-pandemic time series. Notably, the predicted models for total surgical volume, time-sensitive surgeries, and ambulatory diagnostic procedures had an excellent adjustment. Conversely, the models for urgent and elective surgeries presented a less adequate adjustment, denoting the possible need for covariate inclusion (Appendix Figure in S1 Appendix).

**Table 3:** Number of procedures for each 1000 private insurance beneficiaries ≥15 years-old estimated by the SARIMA model in 2020 and 2021.

		Estimated	IC (80%)	IC (95%)
**Total surgical volume** ^a^	Q1/20	29.90	28.69–31.11	28.05–31.75
	Q2/20	33.12	31.91–34.33	31.27–34.97
	Q3/20	31.39	30.18–32.6	29,54–33.24
	Q4/20	29.95	28.74–31.16	28.1–31.81
	Q1/21	31.39	30.1–32.68	29.42–33.37
	Q2/21	33.50	32.21–34.79	31.52–35.47
	Q3/21	32.42	31.12–33.71	30.44–34.39
	Q4/21	30.77	29.48–32.06	28.79–32.74
**Urgent surgeries** ^b^	Q1/20	4.55	4.01–5.09	3.72–5.37
	Q2/20	4.63	4.06–5.2	3.76–5.5
	Q3/20	4.66	4.07–5.26	3.75–5.57
	Q4/20	4.60	3.98–5.22	3.65–5.55
	Q1/21	4.75	4–5.5	3.6–5.9
	Q2/21	4.84	4.06–5.61	3.66–6.01
	Q3/21	4.87	4.08–5.66	3.66–6.08
	Q4/21	4.80	3.99–5.61	3.56–6.04
**Elective surgeries** ^c^	Q1/20	0.39	0.35–0.43	0.33–0.45
	Q2/20	0.42	0.38–0.46	0.36–0.48
	Q3/20	0.40	0.36–0.45	0.34–0.47
	Q4/20	0.38	0.34–0.42	0.32–0.44
	Q1/21	0.40	0.35–0.45	0.33–0.48
	Q2/21	0.43	0.38–0.48	0.36–0.51
	Q3/21	0.42	0.37–0.46	0.34–0.49
	Q4/21	0.39	0.35–0.44	0.32–0.47
**Time-sensitive surgeries** ^d^	Q1/20	0.52	0.38–0.66	0.3–0.74
	Q2/20	0.54	0.47–0.6	0.44–0.64
	Q3/20	0.55	0.46–0.64	0.41–0.69
	Q4/20	0.57	0.46–0.68	0.4–0.74
	Q1/21	0.57	0.54–0.6	0.52–0.62
	Q2/21	0.58	0.45–0.71	0.39–0.78
	Q3/21	0.59	0.55–0.64	0.52–0.66
	Q4/21	0.60	0.55–0.66	0.52–0.69
**Ambulatory diagnostic procedures** ^e^	Q1/20	30.84	29.74–31.95	29.16–32.53
	Q2/20	31.17	29.61–32.73	28.78–33.55
	Q3/20	29.76	27.85–31.67	26.84–32.68
	Q4/20	26.20	23.99–28.4	22.83–29.57
	Q1/21	29.00	25.88–32.11	24.23–33.76
	Q2/21	29.32	25.5–33.14	23.48–35.16
	Q3/21	27.91	23.51–32.32	21.17–34.66
	Q4/21	24.35	19.42–29.28	16.81–31.89

^a^ Estimated values by the SARIMA model (0,0,0) (1,1,0)

^b^ Holt-Winters allowed for temporal series modeling by exponential smoothing

^c^ Holt-Winters allowed for temporal series modeling by exponential smoothing

^d^ Estimated values by the SARIMA model (0,1,0) (0,1,0)

^e^ Estimated values by the SARIMA model (0,1,0) (0,1,0)

Q1 = 1^st^ quarter; Q2 = 2^nd^ quarter; Q3 = 3^rd^ quarter; Q4 = 4^th^ quarter; CI = confidence interval; ADP = ambulatory diagnostic procedures. Note: Urgent surgeries = obstetric procedures + femur fracture; elective surgeries = bariatric surgery + arthroplasty revision; time-sensitive surgeries = breast cancer surgery + uterine cervical cancer surgery + colon and rectal cancer surgery + prostate cancer surgery; ADPs = bronchoscopy + upper endoscopy/esophagogastroduodenoscopy + colonoscopy.

The 2020 and 2021 backlogs were estimated based on the difference between predicted values in the temporal series and those obtained from the ANS’s database. As described in the Methods, such differences were modeled based on generalized mixed models with gamma distribution and the observed coefficients for each procedure category are described in table Table 4. As such, the estimated backlogs were 134.385,64 for total surgical procedures, 2.634,34 for bariatric surgeries and arthroplasty revision (elective surgeries), 2.845,61 for oncologic (time-sensitive) surgeries, and 304.193,99 for ambulatory diagnostic procedures. The estimated time required to make up for such backlogs in the absence of external interfering factors would be 6.9 quarters (1.7 years) for elective surgeries, 27 quarters (6.8 years) for ambulatory diagnostic procedures, and 63.5 quarters (15.9 years) for time-sensitive surgeries.

**Table 4:** Generalized linear models with gamma distribution for backlog estimation.

	Coefficient	CI (Coef,95%)	p-value
Intercept	1.197	[-2.616; 5.01]	0.538
Type of surgery * time (quarters)			
ADP * periodo	-0.262	[-0.456; -0.067]	0.008
Time-sensitive * periodo	0.048	[-0.147; 0.243]	0.630
Elective * period	-0.173	[-0.368; 0.022]	0.082
Type of surgery			
Ambulatory diagnostic procedures	5.875	[0.484; 11.267]	0.033
Time-sensitive	-4.218	[-9.609; 1.174]	0.125
Elective	Reference		

*Note: Urgent surgeries = obstetric procedures + femur fracture; elective surgeries = bariatric surgery + arthroplasty revision; time-sensitive surgeries = breast cancer surgery + uterine cervical cancer surgery + colon and rectal cancer surgery + prostate cancer surgery; Ambulatory diagnostic procedures = bronchoscopy + upper endoscopy/esophagogastroduodenoscopy + colonoscopy.

## 4. Discussion

To our knowledge, this is the first report to document the impact of the COVID-19 pandemic on the Brazilian supplementary healthcare system’s surgical activity. A rapid and marked drop in the number of all studied surgical categories and ambulatory diagnostic procedures was observed in the first quarter of 2020, coincident with the rapid increase in COVID-19 cases. The impact over the time series, however, varied among the different procedure categories analyzed, with bariatric surgeries and arthroplasty revisions returning to pre-pandemic levels by the end of the studied period despite the onset of a new COVID-19 wave. Conversely, time-sensitive surgeries and ambulatory diagnostic procedures remained below their historical averages on the second quarter of 2021, thereby reflecting on the much higher time frame required to eliminate/make up for their backlogs.

The outbreak of the COVID-19 pandemic led to a rearrangement of healthcare systems worldwide. Given the urgent need to redistribute available resources to ensure adequate support in the form of hospital beds, equipment, medications, and human resources, a drastic reduction in surgical volume was document worldwide, with some countries reporting >75% reductions during the first wave of the pandemic in 2020 [15].

In addition, several restrictive policies were imposed upon the population to reduce their circulation, which may have contributed to a lower incidence of trauma and trauma-related surgical procedures. Such hypothesis is supported by reports demonstrating a reduction in traffic accidents [16, 17] and a nearly 20% reduction in trauma-related surgeries in the Brazilian public healthcare system in 2020 [3]. Put into this perspective, enforced social distancing measures may help explain why femur fracture presented a steep downward trend coincident with the onset of the pandemic, and why numbers climbed back to baseline values in 2021 as isolation measures were lifted. Notably, the 4.6% reduction in urgent surgical volume in the Brazilian supplementary health system is in line with the 6% reduction in urgent surgeries reported in the country’s public system in 2020 [4].

However, the reduction seen in urgent surgical volume also reflected a significant decrease in obstetrical procedures. Indeed, we observed a progressive downward trend in the obstetrical activity throughout the studied period, which does not seem to have happened by chance. This phenomenon was also observed in the Brazilian public system [3], as well as in Europe [18], and in the USA [19], likely reflecting complex and multifactorial issues involving perception of risks in case of COVID-19 infection, as well as pandemic-related social/behavioral changes.

Restrictive public health policies also seem to have influenced the number of elective and time-sensitive surgeries, and ambulatory diagnostic procedures. In that sense, Truche et al. have shown that the stricter the social distancing measures, the greater their negative impact on surgical activity in the Brazilian public health system [4]. There was an important drop in elective surgeries and screening procedures/exams in the first quarter of 2020, which remained low throughout the entire year in the public health system [3]. We observed a similar (albeit less intense) behavior for ambulatory diagnostic procedures and time-sensitive surgeries in the supplementary healthcare system, but the trends in elective surgical activity differed quite substantially.

Bariatric surgeries and arthroplasty revisions presented a drastic reduction in the first quarter of 2020, following the onset of the pandemic and new recommendation by the ANS. Their volumes, however, gained traction in the second quarter and, subsequently, fluctuated in higher levels than those observed at the beginning of the pandemic, finally reaching pre-pandemic levels towards the end of the studied period despite increasing numbers of new COVID-19 cases. This has contributed for the relatively low (14.2%) drop in elective surgical volume observed in 2020, compared to a 69% reduction observed in the public system (being 59.7% among small-medium surgical procedures) [3, 4]. Such substantial difference is likely multifactorial and may have resulted from the fact that our temporal series included only bariatric surgeries and arthroplasty revisions in the elective surgery category, but conjunctural and structural factors related to each healthcare system cannot be overlooked.

Indeed, there are dramatic differences between the Brazilian public and supplementary/private healthcare systems, with the latter historically presenting (i) a higher number of hospital beds per number of beneficiaries; (ii) a proportionately larger human resource contingency; (iii) a much lower bureaucratic burden pertaining to the acquisition of supplies; and (iv) a higher operational efficiency. In fact, the operational expertise of several large players in the supplementary health system was paramount during the pandemic, and even benefited the public system by strengthening public-private partnerships [2]. Hence, the swift response implemented in the supplementary system (eg. reallocation of available services and personnel; prompt acquisition of PPE and other equipment/resources; and systematic preoperative testing), allowed private hospitals to resume their surgical activity despite an increasing number of new COVID-19 cases.

From an economic standpoint, such rapid response allowed private hospitals to reverse by 2021 some of the financial losses caused by the massive canceling of elective surgeries in the beginning of the pandemic. From an assistance standpoint, this was key to keep the elective surgical backlog somewhat contained. Unfortunately, that was not the case for time-sensitive surgeries (comprised primarily of oncologic procedures), which remained far below historical values throughout the whole pandemic period. It is possible that this trajectory may have been influenced by an overall decrease in new diagnosis of cancer cases due to reduced screening. This is further corroborated by the significant drop in ambulatory diagnostic procedures (upper endoscopy, colonoscopy, and bronchoscopy) observed in the present investigation following the start of the pandemic. Similarly, there was a significant (>40%) reduction in screening procedures and a 35% drop in notification of new cancer cases in the Brazilian public healthcare system in 2020, corresponding to approximately 15,000 non-diagnosed new cases per month [3, 20].

The calculus to estimate the time interval necessary to make up for the procedural backlogs, while theoretical, serves to illustrate the severity of the situation. It is most likely that time-sensitive surgeries which were not performed during the pandemic will not be happening at all, so the true impact of delayed ambulatory diagnostic procedures and early surgical treatment of oncologic patients is likely still to be felt in the short- to mid-term. As for bariatric surgeries and arthroplasty revisions, it is possible that the estimated ‘make-up interval’ (i.e., 1.7 years) is more in line with reality. Even so, such estimation is based solely on the historical growth of the sector and does not account for (i) the fluctuation on the number of available hospital beds over time, (ii) the availability of resources, and (iii) the changes in surgical flow that took place during the pandemic.

Therefore, dealing with an increased surgical backlog and their short and mid-term consequences poses an additional challenge to the sustainability of the private healthcare system. In Brazil, as in the USA, substantial financial losses triggered by the pandemic in 2020 helped to further consolidate the healthcare sector and contributed to more widespread adoption of bundle payments [21–23]. In this context, promoting operational efficiency with well-established surgical programs such as the *Enhanced Recovery After Surgery (ERAS)* and the *Optimized Surgery in Latin America (Cirurgia Otimizada para América Latina*, *COPAL)* [24, 25] becomes paramount not only to counteract surgical backlog, but also to ensure the viability of the system as a whole.

The present investigation has several limitations. First, the ANS’s databank is made of data directly reported by private healthcare institutions on a quarterly basis. As such, our temporal series is limited to 4 datapoints per year, which affects the SARIMA model and contributes to the wide CIs observed in our surgical volume estimates for 2020 and 2021 (had the pandemic not occurred). Secondly, although the *total* surgical activity is systematically reported every quarter, only a few specific surgical categories are reported separately. Hence, we opted to analyse the surgical procedures reported by the ANS, and subsequently group them into our studied categories (elective, urgent, and time-sensitive). Therefore, caution is recommended while interpreting the findings. For instance, since only bariatric surgeries and arthroplasty revisions were included in our ‘elective surgery’ category, our findings pertaining to this category may not be generalizable to all other elective surgical procedures. Finally, pre-pandemic numbers of ambulatory diagnostic procedures may have been inflated by resource overuse and not necessarily reflect the true necessity of the population of beneficiaries. On a smaller scale, this could also be true for some elective surgical procedures. Although we reckon it would be desirable to know the percentage of overuse in order to calculate the true procedural backlog, such analysis is not feasible based solely on the ANS database.

In conclusion, the COVID-19 pandemic resulted in a significant reduction in surgical activity in the Brazilian private healthcare system in 2020 and 2021. Although some recovery was noted in elective surgeries during the pandemic (despite an increasing number of new COVID-19 cases), thereby mitigating their backlog, a substantial amount of ambulatory diagnostic and time-sensitive surgical procedures ceased to be carried out, and the consequences of such delays are still to be felt in the mid- to long-term.

## Supporting information

S1 Data (XLSX)

S1 Appendix (DOCX)
