# Supplementary material for: Impact of COVID-19 pandemic on surgical activity in the Brazilian private healthcare system
Source: PLoS One. 2023 Dec 14;18(12):e0289032. doi: 10.1371/journal.pone.0289032 (PMC10720996; doi:10.1371/journal.pone.0289032)
Supplement: S1 Appendix — (DOCX) [file pone.0289032.s002.docx]

**Impact of COVID-19 pandemic on surgical volume of the Brazilian private healthcare system**

Luiza Helena Degani Costa^1.2*^, Barbara Yepes Pereira^1^, Isabela Queiros Castro^3^, Heitor Werneck^4^. Glenio B. Mizubuti^5^, Luiz Fernando dos Reis Falcão^2.6^

1. Hospital Israelita Albert Einstein – Faculdade Israelita de Ciências da Saúde Albert Einstein. São Paulo, SP, Brazil
2. Centro Universitário São Camilo, São Paulo, SP, Brazil
3. Hospital Israelita Albert Einstein – Departament of Biostatistics
4. Agência Nacional de Saúde Suplementar, Rio de Janeiro, RJ, Brazil
5. Kingston Health Sciences Center, Queen's University, Kingston, ON, Canada
6. Universidade Federal de São Paulo – Escola Paulista de Medicina (UNIFESP-EPM), São Paulo, SP, Brazil

**Correspondence to:**

Luiza Helena Degani-Costa. MD PhD

Pulmonologist and Professor of Internal Medicine

Faculdade Israelita de Ciências da Saúde Albert Einstein - Hospital Israelita Albert Einstein

Rua Comendador Elias Jafet. 755 - Morumbi. São Paulo - SP. São Paulo - SP. 05653-000

[luiza.falcao@einstein.br](mailto:luiza.falcao@einstein.br) / luizadeg@gmail.com

| **Table of contents** | |  |
| --- | --- | --- |
| **Tables:** |  |  |
| Appendix table 1 | Quarterly surgical and diagnostic procedures throughout 2020 and 2021 as a percentage of volumes recorded in 2019. | **Page 2** |
| Appendix table 2 | Quarterly Urgent. Elective. and Time-sensitive procedures throughout 2020 and 2021 as a percentage of volumes recorded in 2019. | **Page 2** |
| **Figure** | SARIMA model adjustments for (A) Total surgical volume; (B) Urgent surgeries; (C) Elective surgeries; (D) Time-sensitive surgeries; and (E) Ambulatory diagnostic procedures. | **Page 3** |

.

**Tables:**

**Appendix Table 1. Quarterly surgical and diagnostic procedures throughout 2020 and 2021 as a percentage of volumes recorded in 2019.**

|  | | | |
| --- | --- | --- | --- |
|  | | **Total surgical activity** | **Ambulatory diagnostic procedures** |
| 2020 | Q1 | 70.8% | 39.3% |
|  | Q2 | 71.5% | 60.5% |
|  | Q3 | 81.9% | 79.6% |
|  | Q4 | 81.7% | 83.3% |
| 2021 | Q1 | 73.1% | 67.2% |
|  | Q2 | 84.6% | 79.7% |

The color scale (green → yellow → orange → red) and their various shades mark the size of the percent change in quarterly surgical/procedural volumes in 2020 and 2021 compared to the same quarter in 2019. Bright green shades indicate a positive variation, while light green and subsequent yellow, orange and red shades indicate progressively larger negative variations, with the bright red shades marking the greatest negative impact.. *Q1 = 1^st^ quarter, Q2 = 2^nd^ quarter, Q3 = 3^rd^ quarter, Q4 = 4^th^ quarter

**Appendix Table 2. Quarterly Urgent. Elective. and Time-sensitive procedures throughout 2020 and 2021 as a percentage of volumes recorded in 2019.**

|  | | | | |
| --- | --- | --- | --- | --- |
|  | | **Urgent** | **Elective** | **Time-sensitive** |
| 2020 | Q1 | 102.05% | 49.28% | 74.88% |
|  | Q2 | 100.21% | 85.23% | 78.76% |
|  | Q3 | 90.97% | 105.81% | 84.15% |
|  | Q4 | 88.09% | 98.93% | 87.37% |
| 2021 | Q1 | 86.07% | 81.93% | 77.47% |
|  | Q2 | 86.93% | 108.94% | 87.17% |
|  | | | | |

The color scale (green → yellow → orange → red) and their various shades mark the size of the percent change in quarterly surgical/procedural volumes in 2020 and 2021 compared to the same quarter in 2019. Bright green shades indicate a positive variation, while light green and subsequent yellow, orange and red shades indicate progressively larger negative variations, with the bright red shades marking the greatest negative impact. *Q1 = 1^st^ quarter. Q2 = 2^nd^ quarter. Q3 = 3^rd^ quarter. Q4 = 4^th^ quarter. Note: Urgent surgeries = obstetric procedures + femur fracture; elective surgeries = bariatric surgery + arthroplasty revision; time-sensitive surgeries = breast cancer surgery + uterine cervical cancer surgery + colon and rectal cancer surgery + prostate cancer surgery.

**Figure:**

**
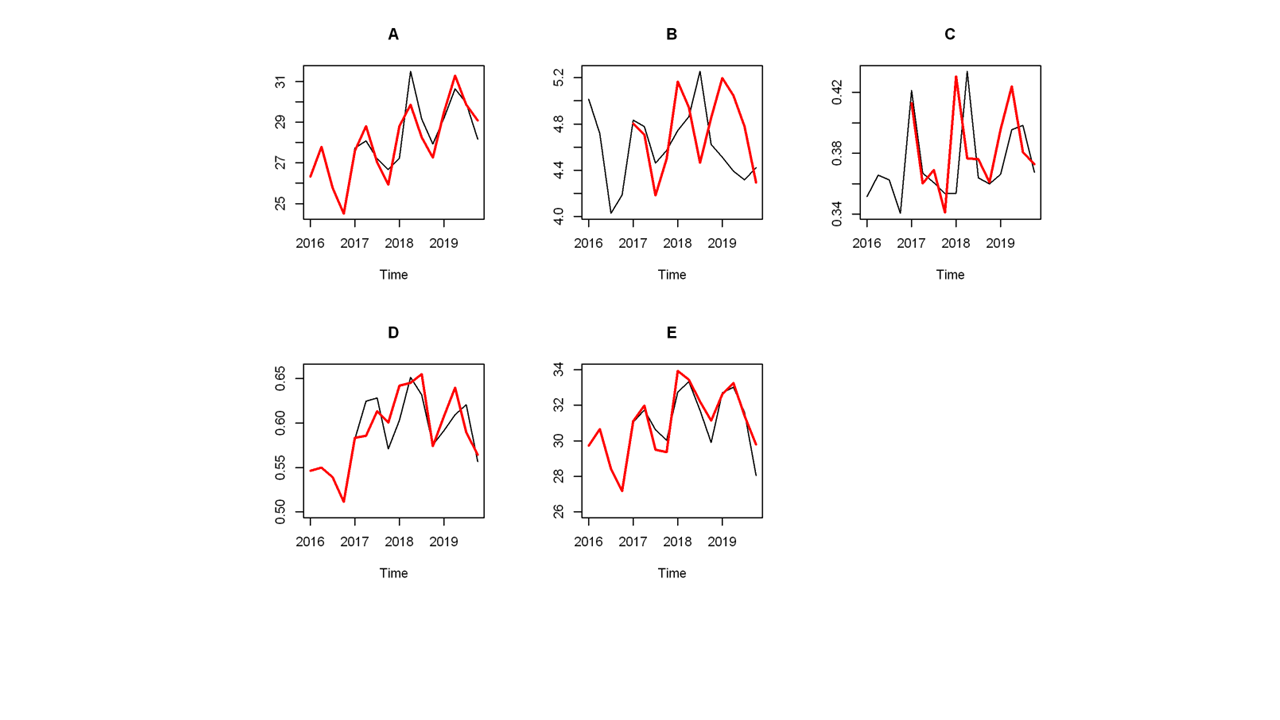
**

**Appendix Figure: SARIMA model adjustments for (A) Total surgical volume; (B) Urgent surgeries; (C) Elective surgeries; (D) Time-sensitive surgeries; and (E) Ambulatory diagnostic procedures.**
